# Are Long-Term Non-Progressors Very Slow Progressors? Insights from the Chelsea and Westminster HIV Cohort, 1988–2010

**DOI:** 10.1371/journal.pone.0029844

**Published:** 2012-02-20

**Authors:** Sundhiya Mandalia, Samantha J. Westrop, Eduard J. Beck, Mark Nelson, Brian G. Gazzard, Nesrina Imami

**Affiliations:** 1 Department of Medicine, Imperial College London, Chelsea and Westminster Hospital, London, United Kingdom; 2 London School of Hygiene & Tropical Medicine, London, United Kingdom; Rush University, United States of America

## Abstract

Define and identify long-term non-progressors (LTNP) and HIV controllers (HIC), and estimate time until disease progression. LTNP are HIV-1^+^ patients who maintain stable CD4^+^ T-cell counts, with no history of opportunistic infection or antiretroviral therapy (ART). HIC are a subset of LTNP who additionally have undetectable viraemia. These individuals may provide insights for prophylactic and therapeutic development. Records of HIV-1^+^ individuals attending Chelsea and Westminster Hospital (1988–2010), were analysed. LTNP were defined as: HIV-1^+^ for >7 years; ART-naïve; no history of opportunistic infection and normal, stable CD4^+^ T-cell counts. MIXED procedure in SAS using random intercept model identified long-term stable CD4^+^ T-cell counts. Survival analysis estimated time since diagnosis until disease progression. Subjects exhibiting long-term stable CD4^+^ T-cell counts with history below the normal range (<450 cells/µl blood) were compared to LTNP whose CD4^+^ T-cell count always remained normal. Within these two groups subjects with HIV-1 RNA load below limit of detection (BLD) were identified. Of 14,227 patients, 1,204 were diagnosed HIV-1^+^ over 7 years ago and were ART-naïve. Estimated time until disease progression for the 20% (239) whose CD4^+^ T-cell counts remained within the normal range, was 6.2 years (IQR: 2.0 to 9.6); significantly longer than 4.0 years (IQR: 1.0 to 7.3) for patients with historical CD4^+^ T-cell count below normal (Logrank chi-squared = 21.26; p<0.001). Within a subpopulation of 312 asymptomatic patients, 50 exhibited long-term stable CD4^+^ T-cell counts. Of these, 13 were LTNP, one of whom met HIC criteria. Of the remaining 37 patients with long-term stable low CD4^+^ T-cell counts, 3 controlled HIV-1 RNA load BLD. Individuals with stable, normal CD4^+^ T-cell counts progressed less rapidly than those with low CD4^+^ T-cell counts. Few LTNP and HIC identified in this and other studies, endorse the need for universal definitions to facilitate comparison.

## Introduction

The majority of HIV-1 infected patients display a gradual decline in peripheral blood CD4^+^ T cells throughout the course of their illness. This is accompanied by excessive immune activation and the sequential loss of immune responses, first to HIV-1, then to other pathogens, allogeneic stimuli and finally mitogens [1], [2]. The rate of disease progression from asymptomatic HIV-1 infection to AIDS varies between patients [3], and determination of factors associated with progression may enable the development of therapeutic or prophylactic vaccines to abrogate disease or prevent infection.

AIDS defining illnesses were originally used as clinical indicators of HIV-1 disease progression, however in 1993 the USA widened their definition of AIDS to include a decline in peripheral blood CD4^+^ T-cell count to less than 200 cells/µl blood or less than 14% of lymphocytes [4]. Some HIV-1^+^ patients are able to maintain stable CD4^+^ T-cell counts within the normal healthy range (between 450–1650 cells/µl blood; local laboratory reference range [5]) for a prolonged length of time and remain asymptomatic without antiretroviral therapy (ART). These patients have been referred to as long-term non-progressors (LTNP). Within this group of atypical patients a few, known as HIV controllers (HIC), suppress HIV-1 replication below the limit of detection of the HIV-1 RNA plasma load assay (BLD; <50 HIV-1 RNA copies/ml plasma) [5], [6]. Studying such atypical patients has the potential to provide insight into the control of HIV-1 infection in the absence of therapeutic intervention [7]. Despite the identification and study of LTNP and HIC in many international single- and multi-centre HIV cohorts, consistent definitions have not been uniformly applied across all studies and sites. Confusion about definitions used also exists in the published literature, resulting in difficulties when comparing results from patient groups within different cohorts.

**Figure 1:**
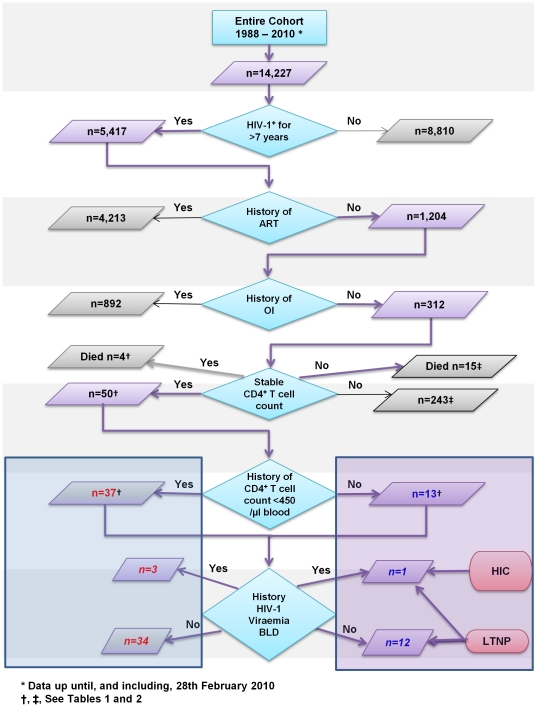
Flow chart detailing the identification of LTNP and patients with long-term stable low CD4^+^ T-cell counts from the Chelsea and Westminster HIV Cohort, during the study period 1988–2010.

The Chelsea and Westminster HIV cohort is the largest single-centre HIV cohort in Europe and is very well documented, with prospective collection of clinical data on all attendees since 1988, facilitating longitudinal analyses. LTNP within the Chelsea and Westminster HIV cohort are defined by a duration of HIV-1 infection longer than 7 years from time of HIV-1^+^ diagnosis, in the absence of ART and clinical symptoms, and the stable maintenance of CD4^+^ T-cell count within the normal reference range used by the laboratory and HIV clinicians during routine clinical follow-up (450–1650 cells/µl blood) [3], [5], [7]. Within this group of patients, a minority, termed HIV controllers (HIC) also control HIV-1 replication to BLD of the conventional HIV-1 RNA load assay; in the past the threshold of viral load detection was 500 HIV-1 RNA copies/ml plasma, but the current assays now have a detection limit of 50 HIV-1 RNA copies/ml plasma [5]. Another group of patients had a history of long-term stable CD4^+^ T-cell counts that on at least one occasion were below the normal range, and these individuals were identified as having long-term stable low CD4^+^ T-cell counts. Amongst this group, patients who exhibited control of HIV-1 RNA load replication to BLD were also identified (Figure 1).

The aims of this study were firstly, to estimate the time until HIV-1 disease progression in groups of patients identified as LTNP during the study period by the application of different selection criteria; secondly to identify the frequency of HIV-1^+^ patients belonging to the Chelsea and Westminster HIV cohort who, at the end of the study period, fulfilled the criteria of LTNP and HIC; and thirdly, to report immunological and virological profiles in the patient groups identified.

## Methods

### Ethics Statement

Data analyses were conducted on anonymised data and this study was approved by the Riverside Research Ethics Committee (now known as London Riverside National Research Ethics Service [NRES] Committee.

### Study sample

Data for each patient in the entire Chelsea and Westminster HIV cohort from 1^st^ January 1988 until 28^th^ February 2010 were extracted from our local database and screened to identify LTNP, HIC, patients with long-term stable low CD4^+^ T-cell counts, and amongst these a subset of patients who also controlled HIV-1 RNA load BLD.

### Cohort selection criteria

The Chelsea and Westminster HIV Cohort LTNP selection criteria were applied to identify patients with documented HIV-1 infection for greater than seven years. The duration of HIV-1 infection was derived from the date of first positive HIV-1 test result or, if this was unavailable prior to enrolment into the local HIV cohort, the earliest clinic visit date was used. The second criterion for patient selection was never receiving ART. Thirdly these patients were screened for history of opportunistic infection (OI). OI was defined as any symptomatic manifestation of HIV-1 illness described in the medical record. Entries of “acute infection”, “persistent generalized lymphadenopathy (PGL)”, and “asymptomatic infection” were not defined as OI. The fourth selection criterion used was patients who had a stable CD4^+^ T-cell count. This was defined as a CD4^+^ T-cell count slope of ≥0 cells/µl blood from entry into cohort until the most recent available CD4^+^ T-cell count. Analyses were performed by grouped 3 monthly time periods because, on average, clinically stable HIV-1^+^ patients are seen at quarterly intervals in our cohort.

### Statistical methods

As longitudinal data on CD4^+^ T-cell count were available, with multiple assessments of CD4^+^ T-cell count on the same patient at different time points, within patient assessments at different time points were expected to be correlated. This correlation needed to be accounted for when selecting analytical methods [8].

The statistical analyses using longitudinal data needed to consider four main characteristics: i) time may be an explanatory variable; ii) repeat CD4^+^ T-cell count measurements for a patient are likely to be correlated; iii) the co-variables may be time-dependent, showing variation through time for a patient; and iv) missing data in the successive CD4^+^ T-cell count measurements may induce a bias. A repeated measures linear mixed model was used to derive time adjusted CD4^+^ T-cell count slope, or rate of change per quarter in CD4^+^ T-cell count since entry to cohort [9].

The MIXED procedure in Statistical Analysis Software (Version 9.1.3; SAS Institute Inc., Cary, NC, USA) was used by fitting values of CD4^+^ T-cell counts from all available results at different time points as a dependent variable grouped into 3 monthly intervals. A random intercept model was fitted using MIXED procedure in SAS. The independent variables included the fixed effects of subjects and clinic visit time points grouped into 3-month intervals. This assumed that the intercept for each patient was random and the effects of CD4^+^ T-cell count changes (slopes) from baseline (at entry into the cohort) were also random and differed between subjects. A compound symmetry covariance matrix was used to model the within patient errors. This assumed that the variances were homogeneous. Correlation existed between two separate measurements, but it was assumed that the correlation was constant regardless of the time between successive CD4^+^ T-cell count measurements [10]. Estimates of CD4^+^ T-cell count slopes were obtained from patient by time interaction. All patients whose CD4^+^ T-cell counts were stable since cohort entry were assumed to show either no change or have an increase in CD4^+^ T-cell count since entry to cohort, indicated by a slope ≥0 cells/µl blood (i.e. no decline in CD4^+^ T-cell count). Those patients whose CD4^+^ T-cell count slope was negative were assumed to have an unstable, declining CD4^+^ T-cell count over time, and were therefore excluded from selection. After excluding patients who had died, a further refined selection criterion was applied to patients who had stable CD4^+^ T-cell counts. Patients were defined as LTNP, who had a stable CD4^+^ T-cell count which continuously remained within the normal range throughout clinical follow-up. These were compared with those patients whose CD4^+^ T-cell counts were stable but had at least one recorded CD4^+^ T-cell count below the normal range (< = 450 cells/µl blood) and referred to as patients with long-term stable low CD4^+^ T-cell counts.

The Mann-Whitney U test was used for between group comparisons of non parametric data while qualitative data are presented as numbers with percentages and compared using the chi-squared test statistic and where appropriate corrected with Yates' correction.

Survival analysis was used to estimate time from HIV-1^+^ diagnosis until disease progression in a group of patients who had been diagnosed HIV-1^+^ for longer than 7 years, and who had not been prescribed ART (n = 1,204). Progression in this group was defined as presentation with symptomatic or AIDS-defining illness or declining CD4^+^ T-cell count during follow-up.

Time from HIV-1^+^ diagnosis until HIV-1 disease progression was estimated in 312 patients, a subset of the initial 1,204, who had been diagnosed HIV-1^+^ for longer than 7 years, had not been prescribed ART and who remained asymptomatic. Progression in this group was assessed through evidence of unstable CD4^+^ T-cell counts during follow up, or death.

Joint probabilities of non-progression for more than 7 years, and remaining ART-naïve, in patients who fulfilled criteria of LTNP but eventually exhibited HIV-1 disease progression, was used to estimate progression time. This was estimated since the date of HIV-1^+^ diagnosis until each of the defined criteria indicative of HIV-1 disease progression. Data were censored at the most recent visit to the clinic. The progression times are estimated and presented stratified by groups of patients whose CD4^+^ T-cell count either remained within, or was outside on at least one occasion, the normal range.

## Results

### Demographic characteristics

From a total of 14,227 HIV-1^+^ patients in the study cohort, 5,417 subjects were identified as being diagnosed HIV-1^+^ for more than 7 years (Figure 1). Of these, 1,204 had never been prescribed ART of whom 19.9% had CD4^+^ T-cell count history that consistently remained within the normal range. There were no significant differences between patients whose CD4^+^ T-cell count remained within the normal range compared to patients whose CD4^+^ T-cell count had been documented below the normal range; in terms of gender (90.8% and 92.3% male respectively; p = 0.515) or median age (39.9 and 40.2 years respectively; p = 0.485). Significantly fewer Caucasians were seen in the group whose CD4^+^ T-cell count remained within normal range compared to patients whose CD4^+^ T-cell count had been recorded on at least one occasion as below the normal range (58.2% and 68.5% respectively; p = 0.003).

Of 1,204 ART-naïve individuals who had been HIV-1^+^ for longer than 7 years, 312 (25.9%) remained asymptomatic, with 258 patients (83%) exhibiting unstable CD4^+^ T-cell counts (Table 1, Figure 1). A significant difference in median highest recorded HIV-1 load in patients who had an unstable CD4^+^ T-cell count was observed when compared to those who exhibited stable CD4^+^ T-cell counts; 39,022 (IQR: 10,935 to 120,580) compared to 12,769 (IQR: 1,547 to 64,902) HIV-1 RNA copies/ml plasma respectively (p = 0.007). No significant differences were seen in immunological parameters investigated between patients with stable and unstable CD4^+^ T-cell counts (Table 1).

**Table 1:** Demographic, immunological and virological parameters of patients with unstable and stable CD4^+^ T-cell counts.

	n = 312			n = 54		
	Unstable CD4^+^ T-cell count	Stable CD4^+^ T-cell count	∧p-value	Long-term stable low CD4^+^ T-cell count	LTNP	∧p-value
	(Total = 258)	(Total = 54)		(Total = 39)	(Total = 15)	
Alive at most	n = 243‡	n = 50†		n = 37†	n = 13†	
recent time point	(Died n = 15)	(Died n = 4)		(Died n = 2)	(Died n = 2)	
**Demographics**						
Gender (%)						
Female	26(10.7)	4(8.0)		3 (8.1)	1 (7.7)	
Male	217(89.3)	46(92.0)	0.751	34 (91.9)	12 (92.3)	0.584
Ethnicity (%)						
Caucasian	157 (64.6)	34 (68.0)		22 (59.5)	12 (92.3)	
Black African	16 (6.6)	2 (4.0)		2 (5.4)	0 (0.0)	
Other	70 (28.8)	14 (28.0)	0.766	13 (35.1)	1 (7.7)	0.090
Median age at last	39.9	42.7		41.7	40.2	
visit, years	(35.2 to 46.3)	(36.8 to 49.9)		(36.4 to 50.6)	(39.2 to 45.4)	
(IQR) [range]	[23.5 to 69.5]	[24.9 to 68.0]	0.078	[24.9 to 67.1]	[29.6 to 68.0]	0.782
HIV risk (%)						
MSM	213 (87.7)	44 (88.0)		34 (91.9)	10 (76.9)	
Heterosexual	28 (11.5)	3 (6.0)		2 (5.4)	1 (7.7)	
Bisexual	2 (0.8)	3 (6.0)	0.021	1 (2.7)	2 (15.4)	0.234
Median time since	10.1	10.2		9.3	11.9	
HIV-1^+^ diagnosis,	(8.3 to 13.7)	(8.1 to 14.2)		(7.6 to 13.0)	(9.4 to 19.4)	
years (IQR) [range]	[7.0 to 23.2]	[7.0 to 24.1]	0.995	[7.0 to 23.7]	[7.4 to 22.3]	0.048
**Immunology**						
Median nadir CD4^+^	303	337	0.404	296	583	
T-cell count, cells/µl	(226 to 415)	(229 to 460)		(194 to 350)	(512 to 675)	
blood (IQR) [range]	[18 to 1055]	[17 to 1043]		[17 to 400]	[460 to 1043]	<0.001
Median most recent	978	1067		946	1222	
CD8^+^ T-cell count,	(714 to 1302)	(755 to 1293)		(712 to 1219)	(1185 to 1624)	
cells/µl blood	[168 to 3255]	[328 to 3168]	0.613	[328 to 1803]	[721 to 3168]	0.015
(IQR) [range]						
Median nadir CD19^+^	90	100		112	94	
B-cell count, cells/µl	(61 to 140)	(59 to 176)		(53 to 187)	(70 to 157)	
blood (IQR) [range]	[4 to 584]	[15 to 348]		[15 to 348]	[41 to 269]	
	n = 147	n = 46	0.354	n = 34	n = 12	0.881
Median nadir	38	52		52	52	
CD16/56 natural killer	(18 to 80)	(24 to 89)		(25 to 90)	(24 to 61)	
cell count, cells/µl	[2 to 362]	[3 to 226]		[11 to 226]	[3 to 191]	
blood (IQR) [range]	n = 129	n = 42	0.218	n = 32	n = 10	0.701
**Virology**						
Median highest	39022	12769		13022	3113	
recorded HIV-1 load,	(10935 to 120580)	(1547 to 64902)		(3424 to 66433)	(1100 to 42238)	
RNA copies/ml	[<50 to 544551]	[<50 to 533774]		[<50 to 533774]	[<50 to 481775]	
plasma (IQR) [range]	n = 130	n = 44	0.007	n = 32	n = 12	0.377

Where data is unavailable for all patients within a group, the number of patients for whom data available is detailed.

‡, † *See* *Figure 1* *for definition of patient groups.*

*∧p-value using Mann-Whitney U test for quantitative data and chi-squared test with Yates' correction for qualitative data.*

### Time until HIV-1 disease progression

Of the 1,204 patients, 239 patients whose CD4^+^ T-cell counts remained within the normal range had an estimated time until HIV-1 disease progression since HIV-1^+^ diagnosis of 6.2 (IQR: 2.0 to 9.6) years, compared to 4.0 (IQR: 1.0 to 7.3) years for the 965 patients who had at least one CD4^+^ T-cell count below the normal range (Logrank chi-squared = 21.26, p<0.001; Table 2).

**Table 2:** Time since HIV-1^+^ diagnosis until disease progression in patients who have been infected with HIV-1 for more than 7 years and who remain symptomless in the absence of ART.

	HIV-1^+^ >7 years, no ART†			HIV-1^+^ >7 years, no ART† or OI†			HIV-1^+^ >7 years, no ART or OI, unstable CD4^+^ T-cell count‡		
	n = 1,204	Time until disease progression#	*p-value*	n = 312	Time until disease progression#	*p-value*	n = 258/312	Time until disease progression#	*p-value*
**CD4^+^ T-cell count ever below the normal range?**									
**No**	**239**	6.2 (2.0 to 9.6)		**110**	9.1 (4.0 to 20.2)		**95**	5.8 (2.3 to 8.6)	
**Yes**	**965**	4.0 (1.0 to 7.3)	*<0.001*	**202**	7.3 (2.3 to 13.3)	*0.054*	**163**	4.6 (1.8 to 8.4)	*0.833*

Patients are stratified according to a history of at least one CD4^+^ T-cell count below the normal range (<450 cells/µl blood).

†, ‡ See Figure 1 for definition of patient groups.

# median (IQR) years.

p-values using the Logrank chi-squared test.

Of the 312 (26%) ART-naïve patients who had been diagnosed HIV-1^+^ for longer than 7 years and remained asymptomatic during the course of their routine HIV-1 follow up, 110 (35.3% of 312) maintained CD4^+^ T-cell counts within the normal range. These patients had an estimated median time until CD4^+^ T-cell count decline (or clinical progression) of 9.1 (IQR: 4.0 to 20.2) years while for the remaining 202 patients who had at least one CD4^+^ T-cell count fall below the normal range this was 7.3 (IQR: 2.3 to 13.3) years (Logrank chi-squared = 3.72, p = 0.054; Table 2).

### Frequency of unstable CD4^+^ T-cell count

Of the 312 ART-naïve patients diagnosed as HIV-1^+^ for longer than 7 years who remained asymptomatic during the course of their routine HIV-1 follow up, 258 (82.7%) had unstable or declining CD4^+^ T-cell counts. Of these 95 (36.8%) had CD4^+^ T-cell counts consistently within the normal range and the estimated median time until HIV-1 disease progression in this group was 5.8 (IQR: 2.3 to 8.6) years, this was similar to the 163 patients (63.2% of 258) who had a history of CD4^+^ T-cell counts below the normal range with an estimated time until HIV-1 disease progression of 4.6 (IQR: 1.8 to 8.4) years (Logrank chi-squared = 0.045, p = 0.833; Table 2).

### Frequency of long-term stable low CD4^+^ T-cell count and LTNP

**Figure 2:**
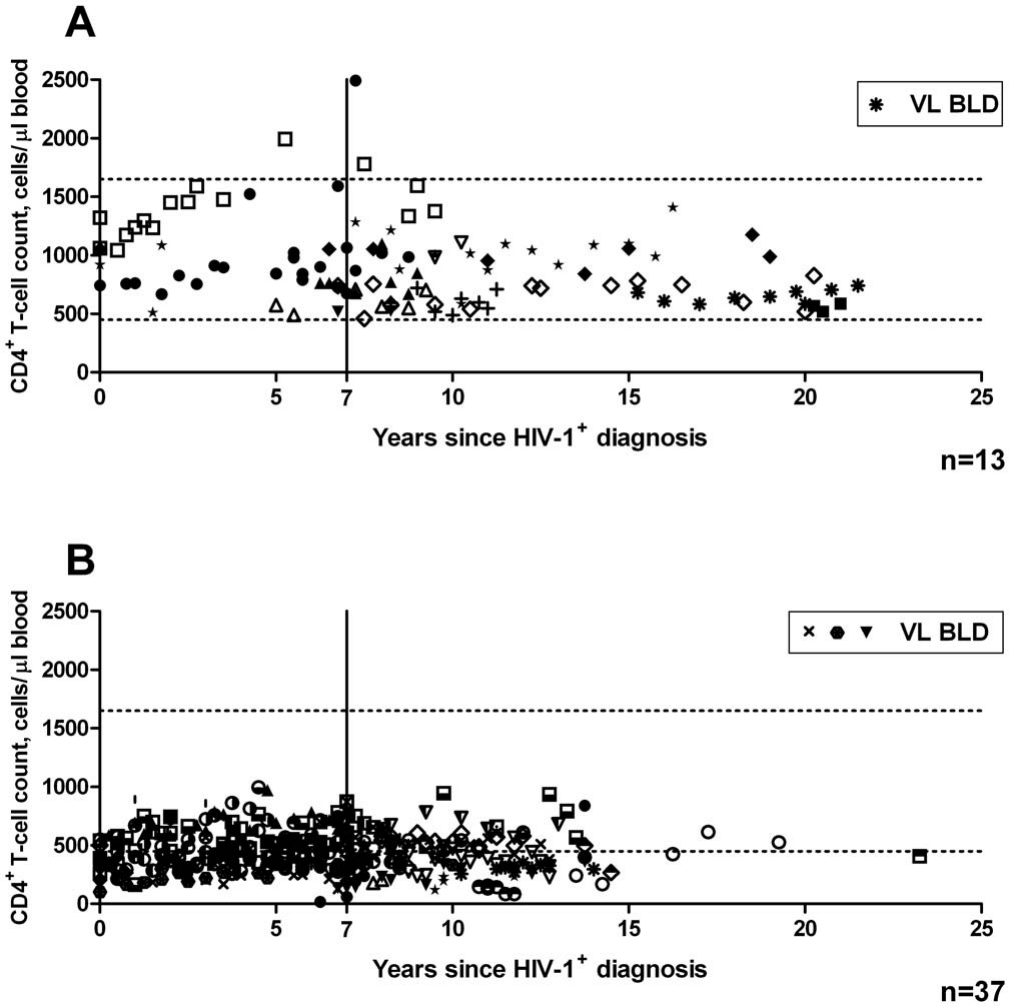
CD4^+^ T-cell counts over the time since HIV-1^+^ diagnosis of (A) 13 LTNP, one of whom fulfilled HIC status, with VLBLD, as detailed in the key and (B) 37 patients with long-term stable low CD4^+^ T-cell count, 3 of whom maintained HIV-1 RNA load to BLD. Dashed horizontal lines indicate the normal healthy range of CD4^+^ T-cell count (450–1650 cells/µl blood), and a solid vertical line shows the time point 7 years post HIV-1^+^ diagnosis. Repeated CD4 T-cell counts are plotted at the time points indicated, where different symbols represent different individual patients.

A total of 50 ART-naïve patients were identified as having long-term stable CD4^+^ T-cell counts. HIV-1 RNA plasma load data from these 50 living patients were investigated, and for four patients this was found to be consistently BLD of the viral load assay used. Of these 50 patients, 13 patients consistently had CD4^+^ T-cell counts within the normal range. These were classified as LTNP (Figure 1), while 1 patient (7.7%) was identified as an HIC. The remaining 37 patients who exhibited long-term stable low CD4^+^ T-cell counts, all had at least one recorded CD4^+^ T-cell count below the normal range. Three of these 37 controlled HIV-1 replication to BLD. CD4^+^ T-cell count profiles over time since HIV-1^+^ diagnosis of the 13 LTNP are depicted in Figure 2A and for those with long-term stable low CD4^+^ T-cell count in Figure 2B. All of these 50 individuals displayed a stable slope of ≥0 cells/µl blood, per quarter.

### Demographic characteristics – comparison of groups of patients with long-term stable low CD4^+^ T-cell count and LTNP

No significant differences were found in gender, ethnicity, age or HIV-1 risk between patients classified as exhibiting long-term stable low CD4^+^ T-cell count and LTNP (Table 1). Duration since HIV-1^+^ diagnosis, nadir CD4^+^ and recent CD8^+^ T-cell counts were significantly higher in LTNP compared to the patients with long-term stable low CD4^+^ T-cell count.

## Discussion

Of the 6,390 HIV-1^+^ patients currently attending our centre for HIV treatment and care, 13 (0.20%) have been identified in this study as LTNP, of whom one (0.02%) was an HIC. Thirty-seven (0.58%) were classified as exhibiting long-term stable low CD4^+^ T-cell counts, of whom 3 (0.05%) were able to control their HIV-1 RNA load replication BLD. This demonstrates that the number of patients fulfilling the criteria used to define LTNP, HIC or long-term stable low CD4^+^ T-cell counts within the Chelsea and Westminster HIV cohort is very small.

**Figure 3:**
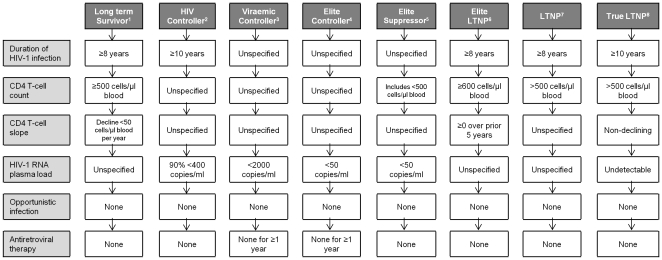
Synonyms used to describe HIV-1^+^ patients exhibiting atypical disease progression. No internationally recognised consensus for terminology currently exists, making comparison between studies difficult. ^1^ [21], [22], ^2^ [5], [6], [19], [23], ^3^ [24], ^4^ [6], [18], [19], [24], ^5^ [6], [25], ^6^ [19], ^7^ [5], [6], [16], [18], [19], [23], [26]–[28], ^8^ [16], [27].

Studies on HIV-1^+^ non-progressors to date have used varying definitions based on duration of HIV-1 infection and CD4^+^ T-cell counts to identify atypical patient groups (Figure 3). In addition, most studies select these atypical patients who maintain their CD4^+^ T-cell count profile within the normal range. This study suggests that by using varying selection criteria, disease progression is very likely in the majority of people living with HIV-1. The patients who had not progressed within the study period are likely to do so, as demonstrated in the analysis of individuals found to have long-term stable low CD4^+^ T-cell counts compared to those with unstable CD4^+^ T-cell counts.

The Chelsea and Westminster HIV Cohort selection criteria included the stringent criterion of a non-declining CD4^+^ T-cell count slope since cohort entry, combined with additional clinical criteria as reported here. Despite having used one of the largest HIV-1^+^ cohorts in Europe to identify these atypical patients, only 50 (0.38%) exhibited maintenance of a long-term stable CD4^+^ T-cell count, of whom only 13 could be confirmed as LTNP. Data on our cohort are routinely collected, and like other observational databases patients can be lost to follow up. Another limitation of observational data from a single centre is that HIV-1^+^ patients can also be managed in other centres and may receive ARV treatment from other clinical care providers, including private practice, which may not be captured in the database of the single centre. The formation of a national centralised observational database would be one strategy to overcome such limitations whilst clinicians are currently reliant on patients' self reporting of treatment, including ART received elsewhere.

Marked differences were observed in some of the immunological and virological markers of HIV-1 disease progression between groups of patients. Current CD4^+^ T-cell count, nadir CD4^+^ T-cell count and plasma HIV-1 RNA load were shown to be statistically different between groups of patients, however no differences were observed in CD8^+^ T-cell, CD19 B-cell or CD16/56 NK cell counts, indicating that the characteristics of HIV-1 disease, in the groups of i) LTNP, ii) patients with long-term stable low CD4^+^ T-cell count and iii) individuals with unstable CD4^+^ T-cell counts, may be independent of the humoral and NK cell-mediated innate immune responses. However, due to the small numbers of individuals in the LTNP and long-term stable low CD4^+^ T-cell count groups, power to detect a statistical significance was low. Small sample sizes decrease accuracy in point estimates used to describe quantitative data, including immunological and virological markers, demonstrated by increased variability. A larger sample size would be required to reduce this variability, providing increased statistical power, sensitive hypothesis tests and decreased variability with narrower confidence intervals. In addition, studies that have investigated factors influencing CD4^+^ T-cell counts have indicated that sex [11], ethnicity [12], age [13] and behavioral (i.e. smoking) [11] factors exert significant effects on CD4^+^ T-cell counts. Such potential effects on the observations reported above could not be performed due to small sample size, and relatively low numbers of women and ethnic diversity in our cohort. Furthermore, the infecting clade of HIV-1 and the geographic origin of infection are likely to impact the course of HIV-1 disease progression. Clade data was only available for 19/50 patients with stable CD4^+^ T-cell count, 16 of whom were infected with clade B and 3 with clade C virus. Of the 13 patients identified as LTNP, clade information was available for 5. Of these, 3 were infected with clade B and 2 with clade C virus, while the infecting viral clade for the remaining 8 LTNP has not yet been tested. Although, the majority of HIV-1^+^ individuals in the UK are infected with clade B virus [14] the infecting clade and viral fitness are important considerations for future work with the cases identified herein.

It is possible that many more PLHIV are LTNP or HIC, but due to the absence of clinical manifestations of disease these individuals have not yet attended an HIV-1 testing facility and consequently have not been diagnosed as HIV-1^+^. The criteria of time from HIV-1 seropositive diagnosis should also be considered as relatively arbitrary, as the date of first HIV-1^+^ test result, or date of first attendance at an HIV clinic, may not be an accurate representation of the date of infection. Instead, the date of first positive HIV-1 test should be considered a tool for healthcare professionals to identify patients exhibiting an unusual course of HIV-1 disease.

Previously published data from our group and others further indicate that disease progression in LTNP is ultimately inevitable, and virological, immunological and genetic factors have all been consistently reported to be associated with rate of disease progression [5], [15], [16]. HIV-1 control is thought to be attributable to immunological, virological and genetic components. The viral control exhibited by LTNP and HIC has been shown to be associated with robust, broad HIV-1-specific CD4^+^ and CD8^+^ T-cell responses [5]–[7]. Many LTNP are HLA-B57^+^, an MHC-class I allele that demonstrates enhanced peptide presentation to cytotoxic CD8^+^ T-cells on the surface of infected CD4^+^ cells [17], [18].

In our opinion, the rate of disease progression in HIV-1^+^ patients should be considered a continuous variable and not discrete. Studying patients at the extreme of this distribution may enable discovery of correlates of HIV-1^+^ disease progression, leading to identification of targets to be manipulated by novel therapeutic approaches, with the ultimate goal of inducing delayed disease progression, retarding ART initiation and alleviating pill burden and toxicity.

Reports from other cohorts where atypical groups of patients have been identified, even though they used different criteria from those stated here, confirm the paucity of PLHIV who could be classified as LTNP. A recent investigation of a large HIV-1 cohort in France identify only 0.4% of their sample as LTNP [19]. The data presented in this and other studies emphasise a need for collaboration with other cohorts when studying such atypical patients, such as work performed within the international GISHEAL consortium, to increase the number of LTNP and HIC who can be identified and studied. In this study we demonstrate that using different criteria to define patient groups result in different estimates in time until disease progression, before even considering functional immunology, genetic and virological factors, further emphasising the need for agreed standardised use of terminology and definitions before more in depth study of these individuals is performed. Figure 3 provides an indication of some of the different criteria used by different groups. Large cohorts of well-defined HIV-1^+^ patients will be essential for future investigation of genetic associations with HIV-1 control and delayed disease progression [6], as the unique immunological and virological responses demonstrated by LTNP and HIC may provide clues towards both the change in disease status of these patients over time, and provide insights for HIV-1 preventive or therapeutic vaccine development, sentiments recently reiterated by Francoise Barre-Sinoussi as part of the preparations for the June 2011 United Nations High Level Meeting in New York [20].
